# Does a patient's health potential affect the social valuation of health services?

**DOI:** 10.1371/journal.pone.0192585

**Published:** 2018-04-24

**Authors:** Jeff Richardson, Angelo Iezzi, Aimee Maxwell

**Affiliations:** Centre for Health Economics, Monash Business School, Monash University, Clayton, Vic, Australia; Harvard Medical School, UNITED STATES

## Abstract

**Background:**

Patients with a permanent impairment may be unable to reach full health. Consequently health services which cure illnesses which are unrelated to the impairment may increase health less than services for patients with no impairment. While it has been argued that this should not lead to discrimination against impaired patients there is little evidence to determine whether this equity-efficiency trade-off is consistent with social values.

**Objectives:**

To measure the effect of permanent impairment upon the social valuation of services for unrelated illnesses.

**Methods:**

Social valuations of services for illnesses associated with mobility, depression or pain were assessed and compared for patients with and without a permanent impairment using the Relative Social Willingness to Pay (RS-WTP) instrument. The maximum valuation of services for impaired patients was also compared with the maximum utility which could be gained when utility was measured using three multi attribute utility instruments.

**Results:**

Curing the illness of impaired patients was valued 8–11 percent less than the cure of patients with no impairment. Discrimination decreased as the severity of the illness increased. Valuation of health states using the utility instruments implied significantly greater discrimination than the social valuations using the RS-WTP instrument.

**Conclusions:**

Health services are valued less highly when a patient’s health potential is impaired. However discrimination is significantly less than would occur if the value of the services were limited to the value of the health state causing the impairment. The argument for disregarding a patient’s limited health potential when resources are allocated therefore receives some support from social valuations but the case for completely equal treatment depends upon additional ethical arguments.

## Introduction

Cost Utility Analyses (CUA) assists the prioritisation of resources by comparing the cost per additional quality adjusted life year (QALY) of different health services where QALYs are calculated as life years times the utility of the relevant health state. Generally, services are recommended when, all else equal, cost per QALY is low as this allows more QALYs to be obtained from the health budget. However in some circumstances there may be a social preference for reducing the QALY gain in order to achieve a fairer distribution of benefits. The significant literature relating to these exceptions has been surveyed by Nord; Gu et al.; Whitty et al. [1–3].

An issue which has received relatively little attention is the potential discrimination against patients with a permanent impairment which reduces their health potential–the best health they can achieve–and therefore the number of QALYs which may be obtained from health services. In the most widely discussed case, a patient with an impaired potential for full health would gain fewer QALYs from an unrelated, life extending treatment than a patient whose additional life years were in full health: life extension times a utility of 1.00 exceeds life extension times a utility less than 1.00. The patient with an impairment would face what has been described as a ‘double jeopardy’ [4]: the disadvantage of the impairment and the additional disadvantage when life saving treatments are prioritised [1, 5–7]. However a similar problem may arise if a permanent impairment prevents a patient from obtaining the same increase in the quality of life from a service as other patients [8–10].

The literature discussing these issues has been primarily concerned with the ethics of discrimination and whether a poorer prognosis because of an unrelated impairment should result in lower priority for patients [11–16]. The ethical debate, reviewed by Nord [1], is summarised in a more recent article by the same author as follows:

‘People with moderate *treatability*, ie moderate potentials for health improvements … should have the same–or much the same–access to health care as those with greater treatability and potentials. They have no less interest in being treated and … it would be unfair to discriminate against them just because the gain that can be provided to them is objectively smaller than the gain that, at the same cost, can be provided to others’ [17].

The present study is concerned with the empirical question of whether social values and preferences are consistent with this view and would result in the equal valuation of health services when they are given to patients who do and do not have an impairment when the services are for illnesses which are unrelated to the impairment and improve the quality, not length of life.

Unlike the better known case of double jeopardy, the effect upon QALYs and the potential for discrimination in this case is ambiguous. If the relationship between health problems is viewed by the public in the same way as it is modelled in the three Health Utility Index (HUI) multi attribute utility instruments (MAUI) then the disutilities of different problems will be multiplicative and a permanent impairment would reduce the QALY gain from the cure of an unrelated illness. For example, if a permanent impairment reduced utility from 1.0 to 0.8 and an unrelated illness halved utility then together the impairment and illness would reduce utility to 0.8×0.5 = 0.4. Curing the illness would increase utility from 0.4 to 0.8, less than the 0.5 improvement if there were no impairment. The underlying assumption in this case is that a patient with lesser health has less to lose from a subsequent unrelated illness and therefore less to gain from its cure.

However a significant number of studies have identified a social preference for prioritising patients in more severe health states. These have been reviewed by several authors [1, 6, 7]. The studies suggest that impaired patients might receive higher priority than other patients with the same treatable illness but no impairment because of the greater severity of their health state. In sum, impaired patients might receive greater or lesser priority as their health gain from the treatment of an unrelated illness is smaller but the severity of their health state is greater than patients with no impairment. Relatively few empirical studies of the issue have been conducted and questions have commonly sought only agreement/disagreement with the principle of health maximisation versus egalitarian/equal distribution of benefit [1, 8, 9]. For exceptions see Patrick et al. [18] Pinto and Abellan-Perpinan [19].

The objective of the present study was to determine social preferences–preferences for the treatment of other people–with respect to this question. Four questions are examined.

*(i) The null hypothesis*: That a permanent impairment will not affect the social valuation of services for unrelated illnesses which affect a person’s quality of life (QoL).

*(ii) The magnitude of discrimination*: If the null hypothesis is rejected does the magnitude of the discrimination between patients with and without a permanent impairment vary with the severity or the type of the unrelated illness.

*(iii) Value ceiling*: Will the valuation of services for unrelated illnesses be limited to the valuation of the patient’s health potential, the health state which patients with a permanent impairment can achieve or will the effect of the impairment be partially or fully mitigated so the valuation of services will not be capped by the valuation of the patient’s health potential.

*(iv) Value versus Utility*: Does discrimination based upon a social valuation of services differ from discrimination based upon individual utilities as measured by three commonly used multi attribute utility instruments (MAUI).

Measurement, methods and the study survey are described in section 2 below. Results are presented and discussed in the subsequent two sections. The study survey was approved by Monash University Human Research Ethics Committee approval ID: CF15/410–2015000200.

## Methods

### Overview

Terminology and the study design are summarised in Table 1.

**Table 1:** Terminology and study design.

**A. TERMINOLOGY**		
RS-WTP	Social values derived from the Relative Social Willingness to Pay instrument	
(social) value	The (social) valuation of services using the RS-WTP	
Task 1 Task 2	RS-WTP allocation tasks in which patient’s health potential is full health (task 1) or an incurable impairment (task 2)	
*	Indicates values obtained from task 2 when health potential is limited	
Case 1…Case 4	Four cases, each consisting of 1 treatable illness, X, and 1 impairment, GEN	
Illness X	Three illnesses associated with (i) mobility; (ii) pain; or (iii) depression. Health services may change the level of severity or cure the illness	
GEN	Three health states which are caused by the genetically determined impairment. They may also occur due to an illness X (i) moderate depression; (ii) moderate pain; (iii) quadriplegia	
SEV 1 … SEV 4	Four severity levels associated with illness X: SEV 1 = slight; SEV 2 = moderate; SEV 3 = severe; SEV 4 = extreme	
Service A	The service in the RS-WTP which moves a patient from a worse level of severity (or death) to a better level of severity	
Service B	The service in the RS-WTP which moves a patient from the higher level of severity to their health potential: full health (task 1), or their value ceiling (task 2)	
Step 1 … Step 4	The four components of task 1 and task 2 which move a patient between levels of severity	
a_i_ b_i_; (a_i_,* b_i_*)	The proportion of the budget of $40,000 allocated to service A (a, a*) or service B (b_i_ b_i_*) at step i	
value ceiling (cap)	The maximum valuation of services which move an impaired patient to their health potential, GEN.	
SF	The shortfall of the valuation of GEN from full health	
**B. STUDY DESIGN**		
	**Survey 1**	**Survey 2**
	**2 Cases each with:** **• One illness X** **• One impairment GEN**	**2 Cases each with:** **• One illness X** **• One impairment GEN**
**Task 1:** **RS-WTP** Health potential: Full health	Case 1: • X = Mobility [SEV 4 … SEV 1] Case 2 • X = Pain [SEV 4 … SEV 1]	Case 3 • X = Depression [SEV 4 … SEV 1] Case 4 • X = Mobility [SEV 4 … SEV 1]
**Task 2:** **RS-WTP** Health potential: Impairment, (GEN)	Case 1: • X = Mobility [SEV 4 … SEV 1] Plus incurable moderate depression Case 2: • X = Pain [SEV 4 … SEV 1] Plus, incurable moderate depression	Case 3: • Depression [SEV 4 … SEV 1] Plus incurable paraplegia Case 4: • X = Mobility [SEV 4 … SEV 1] Plus, incurable moderate pain

Key:

* indicates the presence of a genetic impairment

The social value of services was assessed using the Relative Social Willingness to Pay (RS-WTP) instrument described below. To test the null hypothesis each survey respondent completed two tasks; an RS-WTP valuation of services to a patient with a treatable illness but no impairment (task 1) and an RS-WTP valuation of the same services for the same treatable illness to a patient with a permanent impairment (task2). The null hypothesis implies no significant difference between the valuations in the two tasks. To examine the second study question, the original RS-WTP instrument was modified in the study design to allow the valuation of services as the severity of the illness varied. The third study question–whether the impairment resulted in a value ceiling (or cap) for unrelated services was investigated by comparing the maximum valuation of services in task 2 with the independent valuation of the health states caused by the impairment in task 1 when it was the result of a treatable illness and the patient had no permanent impairment. These methods were used to analyse four separate cases in which the treated illness, X, was associated with either mobility, pain or depression and, in task 2, the permanent impairment was either moderate pain, moderate depression or paraplegia. To examine the fourth study question health states caused by the permanent impairments were mapped into three utility instruments (MAUI) and the shortfall from full health compared with the shortfall derived from the RS-WTP.

### The RS-WTP

The Relative Social Willingness to Pay (RS-WTP) was developed as an alternative to the Person Trade-off (PTO). Like the PTO, ‘values’ are placed upon services which move people from one health state to another. Unlike the PTO it uses the dollar as a measurement metric. Respondents are asked to evaluate services on behalf of society by allocating a fixed budget between two services. The first–service A–saves a patient from death but leaves them in an imperfect health state. The second–service B–takes a second patient from that health state to best health, as defined on the scale. The opportunity cost of funds spent on one service is the reduction in funds spent on the second service. An index number for the social value of the improvement relative to the value of a QALY is obtained by dividing the amount allocated to a service by the total budget. Properties of the RS-WTP and a comparison with the TTO and PTO are given in Richardson et al. [20]. The more general methods of constant sum paired comparisons (CSPC) and ‘budget pie analysis’ have been reviewed by a number of authors [21–24].

In the present study the RS-WTP instrument was modified, as described below, so that the single imperfect health state evaluated in the original RS-WTP, was replaced by 4 severity levels of an illness and the value of services which moved patients from each of these levels to their health potential was calculated from the amount of the budget allocated to the service. Two parallel assessments were conducted. In the first, task 1, service B moved the patient to full health. In the second, task 2, service B moved patients to their health potential as determined by their permanent impairment. In each of the four cases task 1 and task 2 were completed by the same respondent. Study results were based upon a comparison of the budget allocations in the two tasks.

**Fig 1:**
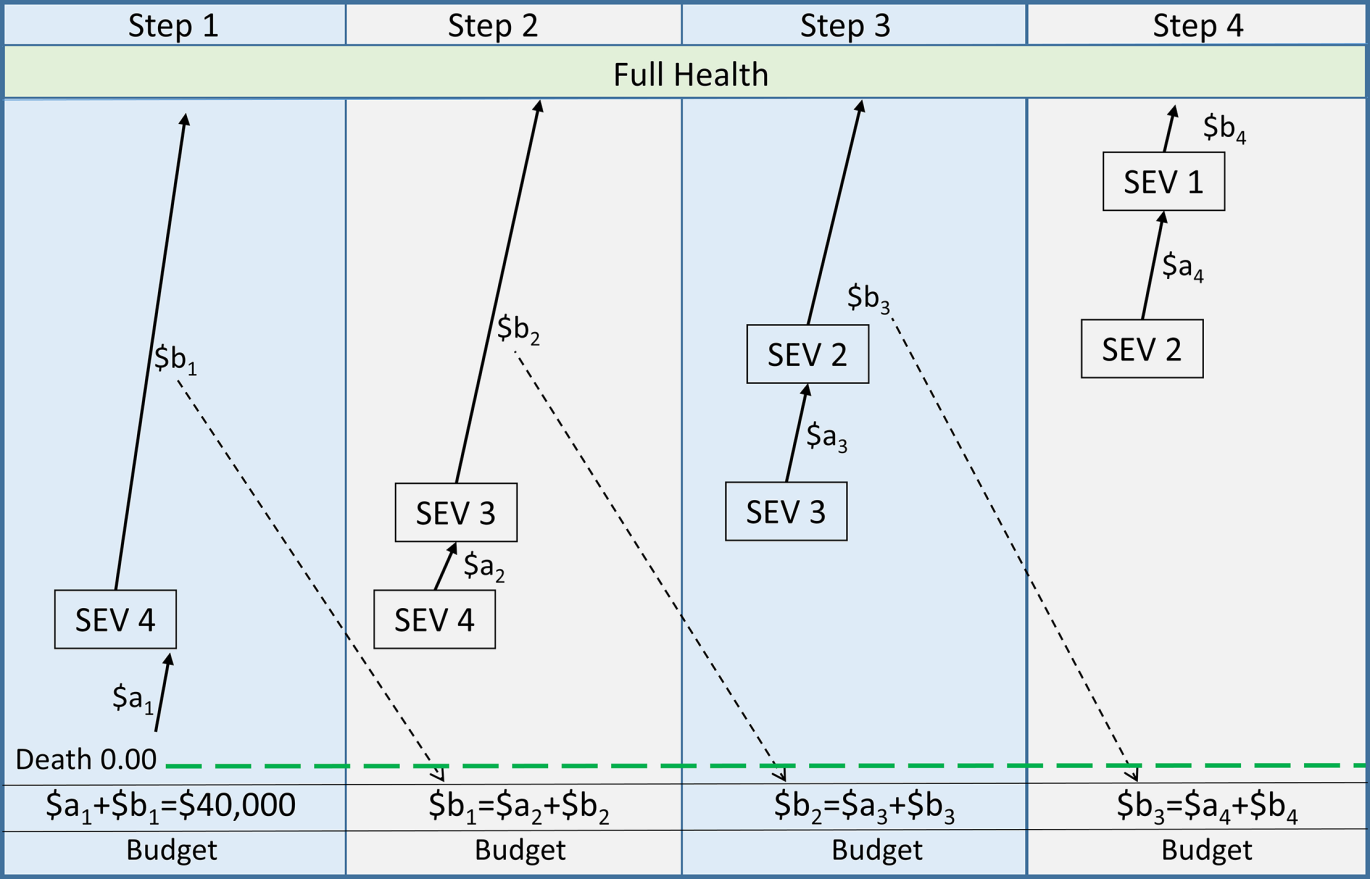
**Multi-step RS-WTP** ^**(a)(b)**^. (a) SEV = severity level; (b) $a_i_ $b_i_ = the dollar allocation to a service, ie the fraction of the initial budget allocated to a service (a_i_, b_i_) times $40,000.

*Task 1*: The modified RS-WTP instrument is shown in Fig 1. In step 1, service A moves a patient from imminent death (numerical value = 0) to severity level 4: SEV 4. Service B moves a second patient from SEV 4 to full health (numerical value = 1.00). The budget of $40,000 is divided between the two services. The budget allocation to service A, divided by the total budget of $40,000 is a_1_; the allocation to service B divided by $40,000 is b_1_. In step 2 the movement from SEV 4 to full health is split into two services; service A moves a patient from SEV 4 to SEV 3, service B from SEV 3 to full health. The budget in this step is the amount which was allocated to the move from SEV 4 to full health in step 1 (ie $40,000.b_1_). In steps 3 and 4 this disaggregation is repeated. The relative valuation of services moving a patient from death to a severity level SEV_j_ is the summation of the valuations of service A $\left(\sum_{i=1}^{j}a_{i}\right)$. The amounts allocated to service B in each case indicate the relative social valuations of a full cure from the four levels of severity and are equal to the dollar allocation to service B divided by $40,000, ie b_1_, b_2_, b_3_, b_4_.

**Fig 2:**
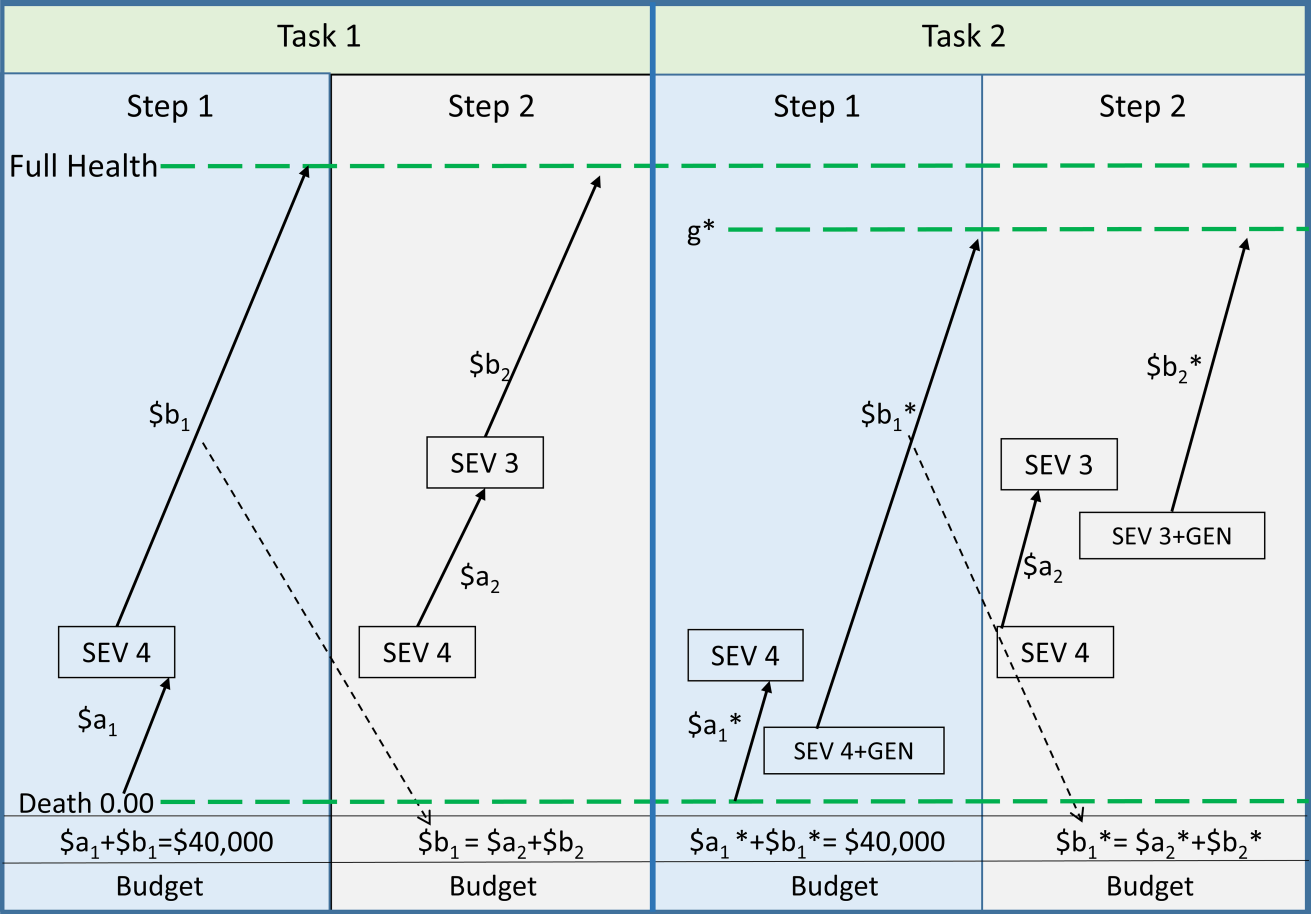
**Multi-step RS-WTP with moderate depression as a permanent impairment in the parallel evaluation**^**(a)(b)**^. (a) SEV = severity level; (b) $a_i_ a_i_*, $b_i_ $b_i_* = the dollar allocation to a service, ie the fraction of the initial budget allocated to a service (a_i_ b_i_) times $40,000.

*Task 2*. To compare the budget allocation to patients with and without an impairment the RS-WTP was further modified. The first two steps of the parallel evaluations are shown in task 2 in Fig 2 and contrasted with the corresponding two steps of task 1 in which full health may be achieved. In task 2 the second patient commences at the lower level of health and can only achieve a health potential determined by the permanent impairment (GEN). Nevertheless, for the reasons given earlier (the severity effect) the maximum valuation of services for patient B, g*, may be equal to 1.00 (the null hypothesis) or, as shown in Fig 2, less than 1.0; that is, it may be subject to a ‘*value ceiling*’, a lesser valuation of a persons’ health potential because of their impairment.

As in task 1, the budgets in steps 2 to step 4 are determined by the allocation to patient B in the previous step, ie by b_1_*, b_2_*, b_3_*, where the asterisk (*) indicates values determined in the context of the impaired patient. For a valid comparison of results from task 1 and task 2 the budget at each step must be equal. As b_i_ and b_i_* may differ, the budgets in task 2, b_i_*, were scaled by the ratio b_i_/b_i_* in steps 2, 3, 4, which equalises the budgets at each step in the two tasks.

#### Aligning value scales

Results of the study were inferred from the comparison of the proportion of the budget allocated to the two services in the two parallel tasks, a_i_ a_i_*; b_i_ b_i_*. However direct comparison could yield invalid results. If respondents believed that health improvement has a different value when a patient has a permanent impairment then the value of a dollar spent in the two tasks differs. In task 1, a_1_+b_1_ = 1: the total dollar expenditures purchase 1 QALY. In task 2, valuation of the same dollar expenditures purchase a health improvement worth g* ie the value of a dollar in task 1 will be equal to the value g* in task 2. To compare the value of services on the same scale, dollars allocated in task 2 were scaled by g*. The maximum valuation of expenditure in task 2 is therefore g* a_1_*+g*b_1_* = g*.

### The surveys

The levels of severity of the 3 illnesses were described using descriptive terms from the EQ-5D-5L MAU instrument as ‘slight’, ‘moderate’, ‘severe’ and ‘extreme’ (or, in the case of mobility, ‘unable to walk: confined to a wheelchair’). To familiarise respondents with the health states they were initially asked to evaluate each health state (the illness and severity level) using a visual analogue scale (VAS).

Task 1 was administered by a talking avatar who introduced the RS-WTP questions in the following way:

‘… Suppose … that you’re a representative on a government committee which decides how to divide a budget between service A and service B…Your committee has a budget of $40,000 which it can divide between the two services according to the benefit they give to patients

Taking *everything* you believe to be important into account divide the $40,000 between service A and service B so that the amounts indicate your view of how Medicare (the Australian health scheme) should value the services.

The survey then proceeded as described in Fig 1.

In task 2 the parallel evaluation was introduced by the avatar in a way which emphasised the permanent impairment.

‘In the following questions the patients receiving service A have the same condition as in the previous four questions but *the patient receiving service B has a genetic condition which causes (statement of the condition)*. *Nothing can cure this*.*’*

The italics text was spoken in a way which parenthesised the incurable impairment. In the visual aid, the box describing the health state of patient B included the impairment in red italicised type: for example ‘*incurable moderate depression*’.

#### Edit criteria

Web-based surveys contain a significant percentage of unreliable answers. Three edit procedures were employed to remove these responses. First, several EQ-5D-5L questions were repeated. Responses were removed when two or more answers did not correspond. Second, responses were removed when respondents could not complete the VAS questions and, third, when answers were judged to be consistently arbitrary as discussed later. Deletions by criteria are given in S1 Table. A comparison of results of the initial allocation to service A severity level 4 with and without deleted responses is given in supplementary S2 Table.

### Analysis

#### The null hypothesis

In step 1 of both task 1 and task 2 the budget is $40,000 and the life saving service which leaves a patient in SEV 4 has the same value: in task 1 it is a_1_ and in task 2 it is g*a_1_*. Therefore a_1_ = g* a_1_* or g* = a_1_/a_1_*. The estimation of g* answers the first study question. If g* = 1 the null hypothesis is confirmed. If g*<1 there is a value ceiling upon expenditures in task 2.

#### Magnitude of discrimination

The value of services to patients with an impairment in task 2 may be calculated at each level of severity by applying the scale factor g* to the budget allocations, b_i_. Scaled values may be compared with values obtained in task 1. This answers the second study question concerning the relationship between discrimination and severity.

#### Value ceiling

The value ceiling, g*, ie the maximum valuation of expenditures upon services for impaired patients, may be compared with the valuation of the health states which are caused by the impairment when they are assessed in task 1 as a result of a treatable illness. If this valuation is less than the value ceiling, g*, then the value of unrelated services are not capped by the valuation of the impaired health state.

#### Value versus utility

The health states (illness plus severity level) caused by the permanent impairments were mapped into the descriptive systems of 3 MAUI, the EQ-5D-5L, SF-6D and HUI 3. Utilities were estimated from the instruments’ algorithms. To address the final study question the shortfall between the average utilities obtained from the MAUI for these health states and full health were compared with the shortfall between the value ceiling, g*, derived from the RS-WTP and full health.

## Results

A total of 662 individuals completed the survey. Data editing led to the removal of 24.8 percent of cases. S2 Table details the reasons. Analysis of the RS-WTP for services, SEV 4, in S2 Table indicates that in most of these cases respondents did not complete the VAS or halved the budget in every allocation irrespective of the illness or the magnitude of the health gain. Nevertheless the inclusion of these data did not make a statistically significant difference to average results (S2 Table). Demographic characteristics of the edited sample are shown in Table 2 and contrasted with the profile of the Australian population. The survey underrepresented males and females aged 18–24 and overrepresented university graduates. Otherwise the sample closely matches the composition of the full population.

**Table 2:** Demographic characteristics and educational attainment of edited survey respondents.

	**A. Survey 1**							
	**18–24**	**25–34**	**35–44**	**45–54**	**55–64**	**65+**	**Total %**	**n**
Males (%)	5.3	7.0	10.2	10.7	8.2	9.0	50.4	123
Females (%)	4.5	6.6	9.8	10.7	7.4	10.7	49.6	121
Total	9.8	13.5	20.1	21.3	15.6	19.7	100	244
Australia (%)	11	19.3	18.2	17.5	15	19		
	**B. Survey 2**							
	**18–24**	**25–34**	**35–44**	**45–54**	**55–64**	**65+**	**Total %**	**n**
Males (%)	4.3	5.5	7.1	11.0	8.7	9.8	46.5	118
Females (%)	4.3	9.8	10.6	9.4	9.1	10.2	53.5	136
Total	8.7	15.4	17.7	20.5	17.7	20.1	100	254
Australia (%)	11	19.3	18.2	17.5	15	19		
	**C. Educational Attainment**							
	**High school**	**Diploma/** **Trade**	**University**	**n**				
Males (%)	25.4	27.5	47.1	244				
Females (%)	24.8	29.9	45.3	254				
Total	25.1	28.7	46.2	498				

(1) 18^+^ source ABS 2015 [25]

The RS-WTP for the treatment of mobility in the absence of a permanent impairment was administered in both surveys. Health state values obtained in the first survey were significantly higher. For reasons detailed in S3 Table, results for case 1 were adjusted by random deletion of records with high RS-WTP for severity level, SEV 4 from case 1 until the mean RS-WTP for SEV 4 was equal to its mean value in survey 2. Analysis was conducted on the parallel assessments of the remaining sample.

Table 3 reports unadjusted RS-WTP values calculated from each health state from the budget allocations to service A. The values from the parallel assessments, task 1 and task 2, are reported in adjoining columns of the table. In task 1 the opportunity cost of service A was a move to full health. In task 2 it was a move to a patient’s limited health potential. The lesser opportunity cost in this latter case results in a greater allocation to service A and greater unadjusted RS-WTP values. Mean values for the four levels differ significantly. In contrast, the table reveals comparatively little variation between health states at the same level of illness severity. The lowest mean value for SEV 4 is for pain; the highest for depression.

**Table 3:** RS-WTP of health states with and without a permanent impairment: unadjusted data^(1)^ ^(2)^ (mean, se).

	Case 1		Case 2		Case 3		Case 4	
	X = Mobility; GEN = Depression		X = Pain; GEN = Depression		X = Depression; GEN = Mobility		X = Mobility; GEN = Pain	
	Potential		Potential		Potential		Potential	
	Task 1	Task 2	Task 1	Task 2	Task 1	Task 2	Task 1	Task 2
	Full health	GEN	Full health	GEN ^(3)^	Full health	GEN ^(3)^	Full health	GEN
SEV 1	0.9 (0.005)	0.92 (0.007)	0.9 (.011)	0.94 *(*.*007)*	0.89 (.010)	0.91 (.009)	0.90 (.014)	0.91 (.007)
SEV 2	0.80 (0.006)	0.84 (0.009)	0.8 (.009)	0.85 *(*.*008)*	0.81 (.013)	0.84 (.01)	0.80 (.011)	0.83 (.009)
SEV 3	0.65 (0.01)	0.7 (0.011)	0.64 (.012)	0.69 *(*.*011)*	0.66 (.014)	0.72 (.011)	0.64 (.014)	0.69 (.012)
SEV 4	0.41 (0.011)	0.46 (0.011)	0.40 (.013)	0.45 *(*.*011)*	0.45 (.014)	0.49 (.011)	0.41 (.014)	0.45 (.012)
n	194	194	292	292	243	243	242	242

(1) The RS-WTP of a health state is the valuation of saving a life and leaving the patient in the defined health state. It is calculated as $\sum_{i=1}^{n}a_{i}\mathrm{or}\sum_{i=1}^{n}a_{i}^{*}$ where a_i_ = the fraction of the budget of $40,000 allocated to service A; and n = the number of levels above death: 1 = SEV 4, 2 = SEV 3, 3 = SEV 2, 4 = SEV 1. The budget in task 2 has been adjusted to equal the budget in task 1. Task 2 allocations have not been rescaled.

(2) Shaded cells give the RS-WTP values for the health states which are caused by the genetic impairment, GEN used in task 2

(3) The genetic impairment is quadriplegia and described as ‘confined to a wheelchair’

### Results of the four study questions

#### Null hypothesis

Mean values for service B are given in Table 4 for the parallel valuations after adjustment of values in task 2 to align value scales. In each of the 16 pairs of results (4 cases times 4 levels of severity) the value of service B is greater for patients with no impairment. The statistical significance of the difference between paired comparisons increases as the severity of the illness increases. The consistency of the result across all comparisons indicates that, with respect to the null hypothesis, a permanent impairment does affect the valuation of services which are unrelated to the impairment. They are valued less.

**Table 4:** RS-WTP valuation of service B^(1)^ with and without a genetic impairment (GEN)^(2)^.

	Case 1				Case 2				Case 3				Case 4				
	Mobility (se): GEN = Depression				Pain (se); GEN = Dep				Depression (se); GEN = Mobility					Mobility (se); GEN = Pain			
	Potential				Potential				Potential					Potential			
	Task 1	Task 2			Task 1	Task 2			Task 1	Task 2	(t)	ratio		Task 1	Task 2		
	Full health	GEN	(t)	ratio	Full health	GEN	(t)	ratio	Full health	GEN				Full health	Pain	(t)	ratio
	b(se)	b*(se)		b*/b	b(se)	b*(se)		b*/b	b(se)	b*(se)		b*/b		b(se)	b*(se)		b*/b
SEV 1	.10 (.01)	.07 (.01)	1.41	0.7	.09 (.006)	.06 (.006)	1.08	0.67	.11 (.01)	.08 (.008)	1.13	0.73		.10 (.008)	.08 (.007)	1.33	0.80
SEV 2	.20 (.01)	.14 (.01)	4.11	0.7	.18 (.013)	.14 (.007)	1.57	0.78	.19 (.013)	.15 (.01)	1.87	0.79		.20 (.011)	.15 (.009)	2.43	0.75
SEV 3	.36 (.01)	.27 (.01)	3.59	0.75	.33 (.012)	.29 (.011)	2.06	0.88	.34 (.014)	.26 (.011)	3.68	0.76		.36 (.014)	.28 (.011)	3.34	0.78
SEV 4	.59 (.01)	.48 (.01)	2.92	0.81	.58 (.013)	.51 (.011)	3.86	0.88	.55 (.014)	.47 (.01)	4.16	0.85		.59 (.014)	.50 (.011)	5.54	0.85

(1) Health state to maximum potential

(2) RS-WTP values for task 2 are scaled by the factor g* = a_1_/a_1_* for reasons described in the text.

#### Magnitude of discrimination

Table 4 also reports, b*/b, the ratio of the budget allocation to patients with and without impairment for each level of severity. The ratio varies from 0.67 to 0.88 with the larger ratios occurring for the most severe health state, SEV 4.

#### Value ceiling

The value ceiling g* = a_1_/a_i_* is reported in Table 5, row 3. It varies from 0.89 to 0.92, a shortfall, SF_1_, of 0.11 and 0.08 respectively from the valuation of full health (row 4). The health states which determine the value ceiling–moderate depression, pain and paraplegia–were assessed in task 1 when the health potential was full health. In Table 3 the relevant valuation is shown by shading. These values are reproduced in Table 5, row 5, (Value 1.GEN). They vary from 0.41 for mobility, SEV 4 to 0.81 for depression, SEV 2, a ‘shortfall’ from full health, SF2, of 0.59 and 0.19 respectively (row 6). In row 7 the shortfall at the value ceiling is compared with the shortfall when the same health state is evaluated independently, SF1/SF2. This indicates that at the value ceiling the shortfall is between 0.20 and 0.57, of the shortfall which would occur if the health states, GEN, were evaluated without reference to impairment. Therefore, while discrimination due to the impairment occurs, it is mitigated relative to the discrimination which would occur if the valuation of services were capped by the valuation of the health state caused by the impairment. In the present cases between 43 and 80 percent of the potential discrimination is mitigated (row 8).

**Table 5:** The value ceiling and mitigation of impairment.

		Case 1	Case 2	Case 3	Case 4
		X = Mobility GEN = Depression	X = Pain GEN = Depression	X = Depression GEN = Mobility	X = Mobility GEN = Pain
**1**	Value a ^(1)^	0.41	0.40	0.45	0.41
**2**	Value a* ^(1)^	0.46	0.45	0.49	0.45
**3**	Value ceiling = g* = a/a*	0.89	0.89	0.92	0.91
**4**	Shortfall (1-g*) = SF1	0.11	0.11	0.08	0.09
**5**	Value 1 GEN = V1.GEN^(2)^	0.81	0.81	0.41	0.80
**6**	Shortfall (1-V1.GEN) = SF2	0.19	0.19	0.59	0.20
**7**	SF1/SF2	0.58	0.58	0.14	0.45
**8**	% Mitigation (1-SF1/SF2).100	0.43	0.43	0.86	0.55

(1) Table 3, service A SEV 4

(2) Table 3, shaded values

#### Value versus utility

Estimated utilities of the health states caused by the genetic impairment in each case are shown in S4 Table for the EQ-5D-5L, HUI 3 and SF-6D along with the health states in the instruments’ descriptive systems which most closely correspond with the impaired health states, GEN. The averages of the 3 utilities are reported in Table 6, row 1 (U.GEN). The shortfall from full health, SF3, is shown in row 2. Rows 4 and 6 are shortfall SF1 and SF2 derived in Table 5 which occur at the value ceiling (SF1) and when the health state caused by the impairment (GEN) is independently valued in stage 1 (SF2). The ratio SF2/SF3 in row 7 therefore compares the shortfall when social value is estimated with no reference to impairment with the (average) shortfall which would occur using the 3 MAU instruments. The greater social valuation for unimpaired patients results in a shortfall which is between 0.76 and 0.99, of the shortfall resulting from the use of the MAUI. This falls to between 16 and 47 percent when the shortfall at the value ceiling is compared with the shortfall derived from the MAUI (SF1/SF3, row 8). That is, the shortfall is significantly reduced when impairment is taken into account.

**Table 6:** Valuation of GEN, the health potential of impaired patients: Utility versus value.

	Case 1	Case 2	Case 3	Case 4
	GEN = Depression	GEN = Depression	GEN = Mobility	GEN = Pain
Utility: U.GEN^(1)^	0.75	0.75	0.51	0.81
Shortfall: (1-U.GEN) = SF3	0.25	0.25	0.49	0.19
Value 1 (GEN) = V1.GEN	0.81	0.81	0.41	0.80
Shortfall: (1-V1.GEN) = SF2	0.19	0.19	0.59	0.20
Value ceiling: g* = a/a*	0.89	0.89	0.92	0.91
Shortfall: (1-a/a*) = SF1	011	0.11	0.08	0.09
SF2/SF3	0.76	0.76	0.80	0.99
SF1/SF3	0.44	0.44	0.16	0.47

(1) Source: S4 Table

## Discussion

Results of the surveys imply that health services to patients with an impairment are valued less than the same services to other patients: social valuations do not imply support for the normative conclusion that a person’s health potential should have no effect upon the valuation of unrelated services. Results are therefore partially consistent with the multiplicative model for combining disutility in which a permanent impairment implies that there is less health to be lost from an unrelated illness and therefore less benefit to be gained from its cure. However there is partial consistency with the ‘severity hypothesis’ as the discrimination against impaired patients declines with the severity of the unrelated illness (Table 4). Discrimination is also significantly mitigated relative to the discrimination that would occur if spending on unrelated services was capped by the independent social valuation of the health states caused by the impairment (Table 5 row 7). The mitigation is greatest in case 3 when the impairment causes the greatest loss of both value and utility. This indicates that results are strongly affected by a consideration of equity which cannot be taken into account directly when services are valued using utility. MAUI seek to measure the strength of preferences by individuals for their own health state. In principle, individuals’ preferences might differ if they were aware that they had a permanent impairment and the re-evaluation of health states might lessen the shortfall from full health after the cure of an unrelated illness. However no utility instrument has attempted to include such an adjustment.

Results strengthen the case for the special treatment of patients with an impairment. If population values found here were to be respected then the discrimination implicit in the use of utility for measuring QoL would need to be largely, but not fully, disregarded. The case for completely disregarding an incurable impairment would need to be based upon additional ethical arguments or for practical reasons as implementing the small degree of discrimination implied by social valuations would be problematical.

Results from the survey may be questioned on several grounds. Respondents may have experienced cognitive difficulty in taking account of the impaired health state while simultaneously evaluating the unrelated services. The survey attempted to minimise this risk. The avatar verbally reminded respondents of the existence of a permanent impairment and the limited health potential of the impaired patient was clearly shown on the visual aids. The statement of the impairment on each visual aid was parenthesised in red italics; that is, there was both verbal and visual reinforcement of the limited capacity for health improvement. For each question the avatar repeated that ‘service B will improve a patient’s health to the ‘*best possible health’* where the patient has no other health problem but remains with the incurable impairment.

To minimise the cognitive burden, very simple health state descriptions were used and people’s interpretation of them was likely to vary. Nevertheless responses clearly distinguished between levels of severity. Each individual’s answer to both of the parallel tasks would be based on their personal understanding of what each question implied for wellbeing and the relationship between the parallel questions was therefore based upon the same interpretation of the health states by each respondent.

The RS-WTP results may also have been affected by framing effects and biases associated with the methodology. However a strength of the study design was that the chief results were derived from parallel RS-WTP questions. Task 1 and task 2 would have the same framing effects and methodological biases. Differences between them can therefore be attributed to the single difference between the tasks, namely the limited health potential of the impaired patient.

The integrity of the survey data may also be challenged for several reasons. First, without the quality control of an interview web-based surveys typically include a non-trivial number of respondents who give ill considered or random results in the minimum possible time in order to obtain the small reward offered by the panel company. Data editing involves some discretionary decisions. While deleting incomplete or inconsistent responses is relatively uncontroversial almost half of the deletions, documented in S1 Table, were made because in every allocation the respondent simply halved the budget irrespective of the illness severity or potential health gain. This improbable result was accompanied by a significantly shorter than average completion time. While the decision was made that these results were unreliable, in principle, people might be providing their preferred allocation. However inclusion of these cases would not significantly alter the pattern of results as data from both task 1 and task 2 would be affected in the same way. Both of the key parameters a_1_ and a_1_* would rise and the effect upon the value ceiling, g* = a_1_/a_1_* would be small.

The more problematical decision was the adjustment of mobility data in the first case to match the results in the fourth case. This is discussed in S1 File adjustments and results compared with TTO valuations of the health states which suggested that RS-WTP values in case 1 were too high. The reason for the discrepancy is unclear but as all else was equal it is likely to reflect an order effect as the two cases were administered first and last. The alternative to adjusting the first mobility data was to average the two sets of results. As with the effect of including deleted data this would have affected results from both task 1 and task 2 and had a relatively small effect upon the comparison of the parallel results.

A further caveat is that results were obtained from a self selected sample, namely those enrolled with a panel company and willing to complete complex questionnaires. The offsetting advantage of this sampling method is that by setting quotas the final sample almost exactly matched the demographic profile of the Australian population and included a cross section of respondents from all educational backgrounds (Table 2, Part C). This does not imply a perfect representation of the population as differences may be associated with other population attributes including ethnic background, religion or location. While there is no clear reason why these attributes should affect the relationship between results from task 1 and task 2 the limitation of the sampling implies that results should be treated with caution and alternative tests of the study questions undertaken.

## Conclusions

Results indicate that people value health services which improve the QoL less highly when they are for patients who have a permanent impairment which is unrelated to the health service. However the implied discrimination is relatively small as compared with the discrimination which would occur if services were valued without reference to a patient’s impairment and, in particular, it is significantly less than the discrimination which would occur if services were valued using utility instruments and the value of the services was limited to the utility of the health state caused by the impairment. Consistent with the concern for severity observed in other studies, the implied discrimination decreases as the severity of the illness increases.

Results therefore give some support to the argument that when resources are allocated there should be little discrimination against impaired patients or that the discrimination should be significantly mitigated relative to the discrimination which would occur if no account is taken of the impairment. However policy conclusions are necessarily inconclusive as ethical considerations may override social preferences.

## Supporting information

S1 File Adjustment of the first mobility sample. (DOCX)

S1 Table Deleted cases by criteria. (DOCX)

S2 Table Comparison of edited and deleted values for Service A, severity level 4. (DOCX)

S3 Table Frequency distribution of RS-WTP values (V) for severity level 4, task 1. (DOCX)

S4 Table Mapping health states causes by impairments into 3 MAUI instruments. (DOCX)
