# Supplementary material for: Does a patient's health potential affect the social valuation of health services?
Source: PLoS One. 2018 Apr 24;13(4):e0192585. doi: 10.1371/journal.pone.0192585 (PMC5918170; doi:10.1371/journal.pone.0192585)
Supplement: S1 File — (DOCX) [file pone.0192585.s001.docx]

**S1 File. Adjustment of the first mobility Sample**

S3 Table reports the frequency distribution of values for severity level 4, SEV 4, for the single problem health states. Significantly fewer respondents assigned low values to severity level 4 for mobility in case 1 in the first survey – ‘mobility 1’ – than for any other case. This resulted in the highest mean RS-WTP for SEV 4 and a significantly higher mean than from the same question in survey 2. TTO utilities were estimated for a parallel study of severity (final row, Table S5) by the transformation of the VAS scores obtained from respondents in both surveys. The TTO for SEV 4 mobility in case 1 was the lowest, not highest, obtained which indicates that the discrepancy in the results was attributable to bias in the first RS-WTP elicitation task, possibly as a result of an order effect as the mobility question was administered first. Consequently an adjustment was carried out which consisted of the random removal of responses to case 1 with high values for SEV 4 for mobility until the mean value equalled the mean for mobility in survey 4. The same cases were removed from the parallel analysis of mobility/depression. Thus the relationship between the allocation to patients with and without a permanent problem was based upon the same sample.
