# Supplementary material for: Does a patient's health potential affect the social valuation of health services?
Source: PLoS One. 2018 Apr 24;13(4):e0192585. doi: 10.1371/journal.pone.0192585 (PMC5918170; doi:10.1371/journal.pone.0192585)
Supplement: S1 Table — (DOCX) [file pone.0192585.s002.docx]

**S1 Table. Deleted cases by criteria**

| **Severity** | **No. of discrepancies in answering the same EQ-5D item** | | **Incomplete or inconsistent VAS** | **Arbitrary data^(1)^** | **Total deleted** | **Percent deleted** | **Edited data (n)** |
| --- | --- | --- | --- | --- | --- | --- | --- |
|  | **Item 2** | **Item 3** |  |  |  |  |  |
| Survey 1 | 20 | 7 | 25 | 30 | 82 | 25.9 | 234 |
| Survey 2 | 8 | 6 | 22 | 46 | 82 | 23.7 | 264 |

(1) For example, the same answer was given for every question, or the budget was always divided evenly irrespective of the illness and severity level or the allocation fell/rose/fell as initial health improved.
