# Supplementary material for: Does a patient's health potential affect the social valuation of health services?
Source: PLoS One. 2018 Apr 24;13(4):e0192585. doi: 10.1371/journal.pone.0192585 (PMC5918170; doi:10.1371/journal.pone.0192585)
Supplement: S2 Table — (DOCX) [file pone.0192585.s003.docx]

**S2 Table. Comparison of edited and deleted values for Service A, severity level 4**

|  | **Survey 1 n=316** | | | **Survey 2 n=346** | | |
| --- | --- | --- | --- | --- | --- | --- |
| **Illness X** | **Percent** | **Case 1** | **Case 2** | **Percent** | **Case 3** | **Case 4** |
|  |  | **X=Mobility GEN=Depression** | **X=Pain GEN=Depression** |  | **X=Depression GEN=Mobility** | **X=Mobility  GEN=Pain** |
|  |  | Mean (se) | Mean (se) |  | Mean (se) | Mean (se) |
| Total data | 100 | 0.48 (0.22) | 0.43 (0.24) | 100 | 0.48 (0.22) | 0.44 (0.22) |
| Deleted data | 26 | 0.56 (0.11) | 0.53 (0.11) | 24 | 0.49 (0.20) | 0.49 (0.21) |
| Edited data | 74 | 0.41 (0.21) | 0.42 (0.23) | 76 | 0.45 (0.22) | 0.41 (0.22) |
