# Supplementary material for: Does a patient's health potential affect the social valuation of health services?
Source: PLoS One. 2018 Apr 24;13(4):e0192585. doi: 10.1371/journal.pone.0192585 (PMC5918170; doi:10.1371/journal.pone.0192585)
Supplement: S3 Table — (DOCX) [file pone.0192585.s004.docx]

**S3 Table. Frequency distribution of RS-WTP values (V) for severity level 4, task 1**

| **Range of RS‑WTP values ($)** | **Mobility 1  Survey 1  (%)** | **Mobility 2 Survey 2  (%)** | **Pain  (%)** | **Depression  (%)** |
| --- | --- | --- | --- | --- |
| 0 | 0 | 6.4 | 6.2 | 6.7 |
| 0<1500 O<V<1,500 | 24.3 | 33.3 | 26.8 | 20.8 |
| 1,500 ≤ V<30,000 | 55.2 | 48.7 | 55.9 | 56.1 |
| 30,000 ≤ V | 20.5 | 11.9 | 11.1 | 16.4 |
|  | 100 | 100 | 100 | 100 |
| TTO: SEV 4 | 0.34 | 0.38 | 0.36 | 0.41 |
