# Supplementary material for: Does a patient's health potential affect the social valuation of health services?
Source: PLoS One. 2018 Apr 24;13(4):e0192585. doi: 10.1371/journal.pone.0192585 (PMC5918170; doi:10.1371/journal.pone.0192585)
Supplement: S4 Table — (DOCX) [file pone.0192585.s005.docx]

**S4_Table Mapping health states caused by impairments into 3 MAU instruments**

| **Health state** | **Moderate Depression** | | **Paraplegia** | | **Moderate Pain** | |
| --- | --- | --- | --- | --- | --- | --- |
|  | **Utility** | **Item level** | **Utility** | **Item level** | **Utility** | **Item level** |
| EQ-5D | 0.85 | Depression Level 3 | 0.34 | Mobility Level 5 | 0.85 | Pain Level 3 |
| HUI 3 | 0.51 | Emotion Level 4 | 0.42 | Ambulation Level 6 | 0.51 | Pain  Level 4 |
| SF-6D | 0.90 | Mental Health Level 3  Role  Level 3 | 0.77 | Physical Level 6 Role level 4 | 0.9 | Pain  Level 4 |
| Average | 0.75 |  | 0.51 |  | 0.75 |  |
